# Decoding the Epigenome of Breast Cancer

**DOI:** 10.3390/ijms26062605

**Published:** 2025-03-13

**Authors:** Elisa Cortellesi, Isabella Savini, Matteo Veneziano, Alessandra Gambacurta, Maria Valeria Catani, Valeria Gasperi

**Affiliations:** 1 Department of Experimental Medicine, Tor Vergata University of Rome, 00133 Rome, Italy; elisa.cortellesi@students.uniroma2.eu (E.C.); savini@uniroma2.it (I.S.); matteo.veneziano@alumni.uniroma2.eu (M.V.); gambacur@uniroma2.it (A.G.); catani@uniroma2.it (M.V.C.); 2 NAST Centre (Nanoscience & Nanotechnology & Innovative Instrumentation), Tor Vergata University of Rome, 00133 Rome, Italy

**Keywords:** epigenetics, breast cancer, histone modifier enzymes, DNA methylation, non-coding RNAs, epi-drugs

## Abstract

Breast cancer (BC) is the most prevalent malignancy among women, characterized by extensive heterogeneity stemming from molecular and genetic alterations. This review explores the intricate epigenetic landscape of BC, highlighting the significant role of epigenetic modifications—particularly DNA methylation, histone modifications, and the influence of non-coding RNAs—in the initiation, progression, and prognosis of the disease. Epigenetic alterations drive crucial processes, including gene expression regulation, cell differentiation, and tumor microenvironment interactions, contributing to tumorigenesis and metastatic potential. Notably, aberrations in DNA methylation patterns, including global hypomethylation and hypermethylation of CpG islands, have been associated with distinct BC subtypes, with implications for early detection and risk assessment. Furthermore, histone modifications, such as acetylation and methylation, affect cancer cell plasticity and aggressiveness by profoundly influencing chromatin dynamics and gene transcription. Finally, non-coding RNAs contribute by modulating epigenetic machinery and gene expression. Despite advances in our knowledge, clinical application of epigenetic therapies in BC is still challenging, often yielding limited efficacy when used alone. However, combining epi-drugs with established treatments shows promise for enhancing therapeutic outcomes. This review underscores the importance of integrating epigenetic insights into personalized BC treatment strategies, emphasizing the potential of epigenetic biomarkers for improving diagnosis, prognosis, and therapeutic response in affected patients.

## 1. Breast Cancer

According to the Global Cancer Observatory of the World Health Organization (WHO), breast cancer (BC) was the most common cancer among women in 157 out of 185 countries in 2022, resulting in approximately 670,000 deaths worldwide [1].

BC, which includes a range of malignancies originating in the mammary gland, is extremely heterogenous given the complex molecular mechanisms leading to tumor initiation and influencing both prognosis and treatment outcomes [2,3]. Indeed, both inter- and intra-tumoral heterogeneity can be recognized; the first one concerns patient-to-patient differences, and the second one is appreciable in cancer cell subpopulations within a patient primary tumor [3].

Based on the origin of cells from which the tumor originates, BC can be categorized in carcinomas, developing from cells lining the terminal lobules and ducts [4], and sarcomas, rare tumors (<1% of primary BC) arising from stromal components of breast, including myofibroblasts and blood vessel cells [5]. In addition, various BC subtypes can be classified according to histopathological features and hormone receptor expression patterns, along with genomic and transcriptomic profiles. Based on histological features, three main BC categories exist: non-invasive (or in situ, confined to the origin site), invasive (or infiltrating, spreading beyond the origin site), and metastatic (spreading to distant organs) cancers, with invasive ductal carcinoma (IDC) and invasive lobular carcinomas (ILC) being the most prevalent types, accounting for about 70% and 15–20% of cases, respectively [6,7].

**Figure 1:**
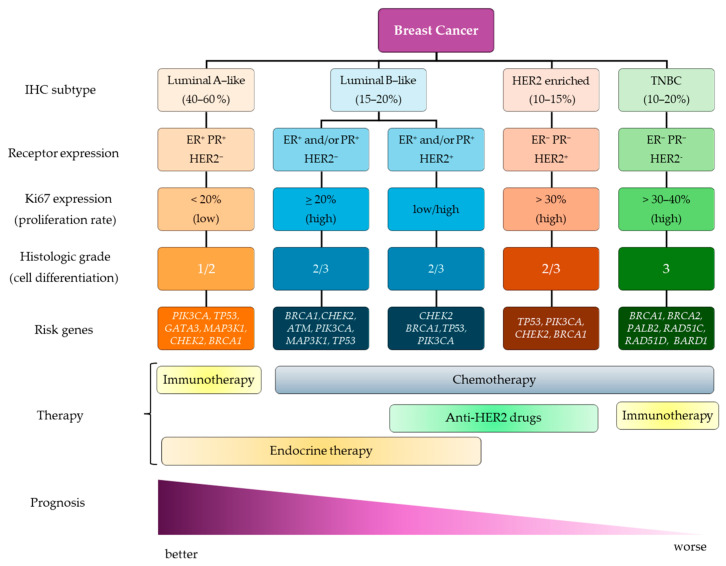
Schematic classification of surrogate intrinsic breast cancer subtypes based on expression of estrogen receptor (ER), progesterone receptor (PR), epidermal growth factor receptor 2 (HER2) and proliferation marker Ki67.

From an immunohistochemical point of view, BC is further classified based on the protein expression of hormone receptors (HR), such as estrogen receptor (ER) and progesterone receptor (PR), as well as of epidermal growth factor receptor 2 (HER2, encoded by the *ERBB2* gene), along with the expression of the proliferation marker Ki67. On this basis, four major surrogate intrinsic subtypes are commonly and widely recognized (Figure 1): (i) luminal A-like (ER^+^, PR^+^, HER2^−^, Ki67^low^); (ii) luminal B-like (ER^+^ and/or PR^+^, HER2^+/−^, Ki67^low/high^); (iii) HER2-enriched (ER^−^, PR^−^, HER2^+^, Ki67^high^); and (iv) triple-negative breast cancer (TNBC; ER^−^, PR^−^, HER2^−^, Ki67^high^), further subclassified into basal-like, claudin-low, mesenchymal, luminal androgen receptor, and immunomodulatory subgroups [8,9].

Approximately 75–80% of BC occurs as sporadic forms, while the remaining cases are hereditary with germline pathogenic variants detected in specific BC-associated genes. *BRCA1* and *BRCA2* are the main high-penetrance genes, conferring about 70% of lifetime risk in mutation carriers [10]. Other genes, such as *PALB2*, *PTEN*, *PIK3CA*, *ATM*, *BARD1*, *CHEK2*, *RAD51C*, *RAD51D*, and *TP53*, have been described for contributing to BC risk, albeit to a lesser extent [11,12,13,14,15,16] (Figure 1). Most of these genes directly and indirectly interact with BRCA1, thus further elevating the risk. Notably, more and more rare monogenic and common polygenic variants are being evaluated for their effects on BC risk [17].

Due to the great heterogeneity, BC treatment varies according to the molecular characteristics and tumor stage, as well as to the responsiveness of the patient [18] (Figure 1). The primary and most common treatment is surgical intervention, either complete or conservative, frequently followed by adjuvant radiotherapy to reduce the risk of local recurrences [19]. Beyond surgery, systemic therapies play a crucial role in BC treatment plans. Aimed at blocking the estrogen proliferative effect on tumor cells, endocrine therapy, such as selective ER modulator (tamoxifen) and aromatase inhibitors (e.g., anastrozole, letrozole, exemestane), represents the cornerstone of therapy for HR^+^ and HER2^−^ BC [20,21]. Recent advancements include the combination of endocrine therapy with immunotherapy based on immune checkpoint inhibitors, namely, the cyclin-dependent kinases 4 and 6 (CDK4/6) inhibitors (palbociclib, ribociclib, abemaciclib), emerging as the most promising therapeutic strategy for advanced luminal BC patients negative for HER2 expression [22]. A multi-targeted approach for both HER2 and ER pathways is crucial for treating HR^+^/HER2^+^ tumors. Combining endocrine therapy with targeted therapies based on specific HER2-targeting antibodies (e.g., trastuzumab, pertuzumab) or pan-HER2 inhibitors (i.e., neratinib) has shown improved efficacy and reduced side effects compared to traditional endocrine therapy and chemotherapy combinations [23,24], thus allowing for the achievement of optimal therapeutic results [25]. Novel therapeutic strategies are actively explored for TNBC patients. While chemotherapy remains the primary approach, immunotherapy using immune checkpoint inhibitors, like programmed death-1/programmed death-ligand 1 (PD-1/PD-L1) inhibitors (pembrolizumab) and cytotoxic T-lymphocyte antigen 4 (CTLA-4) inhibitors (ipilimumab), shows promise for specific patient populations [23,26,27].

Despite significant advances in high-resolution and high-throughput molecular technologies, the precise mechanism(s) underlying tumor transformation in the breast is still not clearly defined [28]; nonetheless, the activation or deactivation of specific genes providing a replicative advantage to BC cells appears to be involved [29], and in this context, epigenetic alterations and chromatin remodeling play a key role, impacting gene expression and genomic stability.

## 2. Epigenetics in BC

Epigenetics refers to “the study of changes in gene function that are mitotically and/or meiotically heritable and that do not entail a change in DNA sequence” [30]. Epigenetic modifications include the post-translational modification (PMT) of histones (particularly methylation and acetylation), DNA methylation, non-coding RNA-mediated modifications, and ATP-dependent chromatin remodeling [31].

Epigenetics plays a pivotal role in BC development and progression. Aberrant epigenetic changes, often triggered by genetic mutations or environmental factors, disrupt the delicate balance of gene expression, activating transcriptional programs, such as those involved in de-differentiation and epithelial-to-mesenchymal transition (EMT), which drive transformation of normal into tumor cells. As cancer advances, the epigenetic landscape continues to evolve. The dynamic interplay between accumulating (epi)genetic alterations and tumor microenvironment shapes the phenotypic heterogeneity of cancer cells and drives tumor progression, including the acquisition of invasive and metastatic properties [32,33]. Beyond their diagnostic potential for early breast cancer detection (as specific epigenetic changes can be detected at very early stages of BC progression), these alterations also offer valuable prognostic and therapeutic insights due to their association with tumor aggressiveness, patient outcomes, and treatment response [34,35].

## 3. DNA Methylation in BC

**Figure 2:**
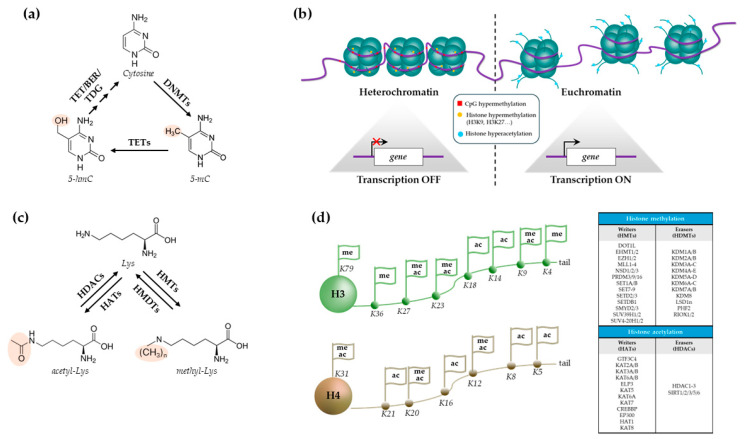
(**a**) Chemical reaction and enzymes involved in the DNA methylation process. (**b**) Impact of DNA and histone modifications on chromatin structure and gene expression. (**c**) Chemical reaction and enzymes involved in the histone acetylation or methylation process. (**d**) Specific lysine (K) residues in H3 and H4 histones that are either acetylated or methylated. All the enzymes involved in lysine modifications are listed in the box. HMT: histone methyltransferase; HMDT: histone demethylase; HAT: histone acetyltransferase; HDAC: histone deacetylase; Lys: lysine; DNMT: DNA methyltransferase; TET: ten-eleven translocation; BER: base excision repair; TDG: thymine-DNA glycosylase; 5-mC: 5-methylcytosine; 5-hmC: 5-hydroxymethylcytosine.

DNA methylation consists of the addition of a methyl group to C-5 position of cytosine within CpG dinucleotides, which are mostly found in the so-called CpG islands present in gene promoters. DNA methyltransferases (DNMTs; DNMT1, DNMT3A, and DNMT3B) catalyze the transfer of the methyl group, thus forming the 5-methylcytosine (5-mC), while removal of methyl groups is driven by ten-eleven translocation (TET) methylcytosine dioxygenases, which hydroxylate 5-mC into 5-hydroxymethylcytosine (5-hmC), which is further oxidized and converted back to cytosine (Figure 2a) [36,37]. When positioned in a gene promoter, DNA methylation represses the transcription of the corresponding gene (Figure 2b).

Aberrant CpG methylation is strongly linked to oncogenic phenotype in nearly all cancer types, including BC [38,39,40]. Genome-wide studies of BC patients have indeed revealed distinct DNA methylation profiles compared to healthy individuals. BC cells typically exhibit global hypomethylation, particularly in the promoters of oncogenes and repetitive regions of the genome. Focal (gene-specific) hypermethylation, especially in the promoters of oncosuppressors, also exists in BC cells, thus repressing transcription [41,42,43,44]. Altered DNA methylation significantly impacts tumor behavior, contributing to genomic instability, aberrant gene expression, and metastasis formation, as summarized in Table 1.

### 3.1. Diagnostic Potential of DNA Methylation in BC Subtyping

Given tumor heterogeneity, several studies have focused on identifying differences in DNA methylation signatures among BC subtypes. Generally, poorly differentiated tumors exhibit higher frequency of methylation of CpGs islands compared to more differentiated ones [68]. Genome-wide CpG methylation changes revealed specific signatures that can overwhelm BC heterogeneity, at least for some subtypes. Indeed, luminal B tumors are characterized by CpG island methylation at promoter regions; basal-like subtypes show high propensity for gene body hypomethylation, whereas luminal A and HER2^+^ subtypes are more heterogenous in terms of DNA methylation patterns [69,70,71]. Alterations in DNA methylation are paralleled by changes in expression of DNMTs [72,73], which are themselves strongly implicated in establishing the epigenetic phenotype of certain BC subtypes [74]: DNMT1 is more overexpressed in TNBC and ER^−^ tumors, DNMT3A is highly expressed in luminal B, HER2^+^, and TNBC subtypes, and DNMT3B is broadly overexpressed across all subtypes [73].

### 3.2. Impact of DNA Methylome on Signaling Pathways and Prognostic Implications

DNA hypermethylation plays a crucial role in the early stages of carcinogenesis, as observed during the transition from healthy breast tissue to DCIS, in contrast to the progression from DCIS to invasive BC, where relatively minimal additional changes have been described [75,76]. These findings therefore highlight the potential of DNA methylation as a valuable biomarker of early detection and risk assessment.

Promoter hypermethylation in early stages of BC development has been reported for genes involved in cell proliferation and differentiation (e.g., *CDKN2A* and *RARβ2*) [53,58]. Furthermore, the finding that some of these genes, such as that encoding for the molecular scaffold protein RASSF1A, exert a more pleiotropic effect (control of cell fate, metabolism, communication, motility and death) [77,78] further reinforces the idea that changes in DNA methylation can serve as potential indicators of malignant behavior and drive the acquisition of cancer phenotype. Notably, DNA promoter hypermethylation frequently targets genes crucial for DNA repair, cell cycle regulation, apoptosis, and suppression of invasion and metastasis. A prime example is *BRCA1*, a gene whose mutations are linked to hereditary BC; even in sporadic BC, *BRCA1* often undergoes epigenetic alterations, specifically promoter hypermethylation, observed in 5–60% of cases [45,46]. This epigenetic silencing impairs DNA repair mechanisms in individuals without a hereditary predisposition, increasing their risk of developing BC. *BRCA1* hypermethylation appears to be mostly associated with BC aggressiveness (grade, Ki67 and HER2 expression, lymph node involvement) [48,79]. In addition, the finding that *BRCA1* hypermethylation has also been found in benign breast lesions (that can later evolve into cancer) and normal tissues adjacent to the tumor site might suggest a potential use of its epigenetic silencing as an early biomarker of genomic instability [48].

During carcinogenesis, hypomethylation of specific coding and non-coding DNA sequences has been documented as well. An illustrative example of an early hypomethylated region is represented by the locus containing the transposable element *long interspersed nuclear element-1* (*LINE-1*) that, under physiologically normal conditions, is in a hypermethylated state, preventing its transposition in the genome. However, hypomethylation occurring in BC reactivates and mobilizes *LINE*-1, thus contributing to genomic instability [66]. DNA hypomethylation has also been observed in genes responsible for tumor immuno-escape, such as *TIM*-3, *PD*-1, *LAG*-3, and *CTLA*-4, encoding for specific immune-checkpoint molecules [80].

Concerning DNA modifier enzymes, DNMT3A and DNMT3B are critical in the early stages of tumor formation [81,82]. Elevated *DNMT3A* expression is instead linked to hypermethylation of *ERα* and *BRCA1* promoters, resulting in their transcriptional silencing and contributing to tumorigenic processes [83]; furthermore, elevated expression of *DNMT3B* and *BRCA1* epigenetic silencing have been associated with poor survival of BC patients [84]. Instead, *DNMT1* is often overexpressed in BC metastatic stages, indicating its involvement in tumor spreading in other organs or tissues [81,82], especially of TNBCs. Remarkably, *DNMT1* is thought to be a cause of TNBC by hypermethylating the promoters of *ER*, several oncosuppressors, and EMT-related target genes (including *CDH1*, encoding for E-cadherin), thus promoting tumor growth, autophagy, and metastasis [51,52,73,85]. As well as *CDH1* promoter silencing, DNMT1 is also responsible for the methylation of *fructose-1,6-biphosphatase* (*FBP1*) promoter, thus contributing to metabolic reprogramming by enhancing glycolytic flux, macromolecule biosynthesis, and ATP production for supporting enhanced cancer cell proliferation [86].

### 3.3. DNA Methylation as a Regulator of Therapy Response

In recent years, there is an increasing number of studies focused on the interplay between DNA methylome and therapy-response; specific DNA methylation profiles are, indeed, associated to therapy-resistance in BC, and meanwhile, changes in DNA methylation patterns occur in response to specific therapeutic drugs.

In this context, DNA methylation signatures could offer a promising avenue for predicting tumor responses to various therapeutic approaches to spare patients who are unlikely to benefit from treatment the burden of unnecessary side effects. Aberrant DNA methylation of specific genes involved in response to hormonal and non-hormonal BC therapy has been described. For instance, hypermethylation-mediated loss of the tumor suppressor *TGFBI* gene correlates with trastuzumab-resistance in HER2^+^ BC subtypes [87]; hypermethylation of *MSH2* and hypomethylation of chemoresistance-related genes (*MDR1*, *MGMT*, *GSTP*, and *UPA*) are linked to doxorubicin-resistance in luminal A and TNBC subtypes [88,89]; while hypermethylation of *CYP1B1* promoter is a highly significant predictor of tamoxifen-resistance as it reduces activation of tamoxifen into its active metabolites in both hormone receptor-positive and hormone receptor-negative subtypes [90].

Conversely, key cell cycle regulators (such as *CDKN2A* and *CCNA1*) appear differentially methylated before and after neoadjuvant chemotherapy (doxorubicin or mitomycin C); therefore, their methylation pattern may predict anthracycline/mitomycin sensitivity, especially for luminal B subtypes [91]. Likewise, significant changes in DNA methylation pattern of four specific genes regulating transcription factor activity, drug metabolism, cell adhesion, and immune functions have been documented in patients before and 5-years after receiving epirubicin and/or paclitaxel-based neoadjuvant therapy [92].

The finding that occurrence and development of a wide range of human cancers, including BC, positively correlate with DNMT expression [93,94,95,96], and that expression is significantly upregulated in tamoxifen-resistant BC tissues compared to sensitive ones [97], suggests these enzymes as promising therapeutic targets for treating cancer, as well as for radio- and chemo-sensitization. To date, several DNMT inhibitors, among which azacytidine (5′-azacytidine) and decitabine (5-aza-2′-deoxycytidine) are the most studied, have been investigated in cancer research. In preclinical ER^+^ and TNBC models, for example, azacytidine and decitabine have been shown to inhibit proliferation, tumor growth, and metastasis (by re-inducing genes regulating apoptosis, cell cycle, stress, and immune signaling pathways) [96,98], as well as to increase sensitivity to chemotherapic drugs [99]; however, clinical studies did not show any benefit for BC patients through the use of DNTM inhibitors alone [100]. Phase II clinical trials evaluating the efficacy of these DNMT inhibitors in combined therapy, such as NCT03295552 (decitabine plus carboplatin for metastatic TNBC), NCT02957968 (decitabine plus pembrolizumab for advanced HER2^−^ BC), and NCT02811497 (azacitidine plus durvalumab for ER^+^/HER2^−^ BC) trials, are still ongoing and/or did not report any available results. Finally, a combined therapy with azacitidine and entinostat (a histone deacetylase inhibitor) has been tested in women with advanced hormone-resistant or TNBC tumors [100]. While in TNBC cohort, no significant change in DNA methylation pattern was observed, in the hormone-resistant cohort, the treatment induced global demethylation, without affecting CpG island promoter methylation. Nonetheless, differential gene expression analysis revealed changes in nearly 200 genes after treatment, of which more than 80% were downregulated. In this specific context, the observed downregulation may be attributable to the effects of DNMT inhibitor-mediated gene body demethylation. Notwithstanding this change in DNA methylation pattern, the combined therapy failed to demonstrate clinical efficacy [100].

## 4. Histone Modifications in BC

Acetylation and methylation of histone (H) tails, particularly of lysine (K) residues, play a pivotal role in regulating chromatin structure and gene expression [101]. Acetylation is catalyzed by histone acetyltransferases (HATs); the acetyl group (derived from acetyl-coenzyme A) neutralizes the K positive charge and breaks the electrostatic interaction between histones and DNA, thus leading to relaxed chromatin state (euchromatin) and positioning of the transcriptional machinery [102]. The reverse deacetylation process is catalyzed by histone deacetylases (HDACs), leading to a more compact and inactive chromatin (heterochromatin) [101,103] (Figure 2b,c).

H3 and H4 histone tails are the primary targets of acetylation/deacetylation cycles (Figure 2d) [104]. Acetylation triggers distinct effects depending on the specific lysine residue modified; for example, H4K16ac is responsible for nucleosome fiber unfolding [105], while H3K27ac distinguishes active from poised enhancers, as well as it shows activity over active promoters [106].

Histone K methylation is catalyzed by histone methyltransferases (HMTs) that use *S*-adenosyl methionine (SAM) as methyl donor to transfer one, two, or three methyl groups to specific residues; the reverse reaction is catalyzed by histone lysine demethylases (HDMTs) (Figure 2d) [101]. H3K4, H3K9, H3K23, H3K27, H3K36, and H3K79 and H4K12, H4K20, and H4K31 are the best-characterized histone methylation sites, where the amino acid can reversibly undergo mono- (me1), di- (me2), and trimethylation (me3) [107]. K residues and methylation state are specified by distinct HMTs and HDMTs. For H3K9, me1→me2 transition is catalyzed by EHMT1/2 (GLP/G9a), me2→me3 transition is catalyzed by SETDB1/2 and SUV39H1/2, and me2→me1 transition is catalyzed KDM3A/3B and KDM4A enzymes; on the other hand, EZH2, the catalytic subunit of polycomb repressive complex 2 (PRC2), and KDM6B are responsible for methylation and demethylation of H3K27, respectively [108]. Unlike acetylation, the impact of methylation on gene expression is dependent on K residues involved and methylation degree: in general, H3K4me3, H3K9me1, H3K36me3, and H3K79me2/3 are found in chromatin regions where actively transcribed genes are present, while H3K27me3 and H3K9me2/3 and H4K20me3 are repressive modifications typically associated with condensed chromatin and silenced genes [109].

During the progression of mammary carcinoma, a complex landscape of alterations in histone acetylation and methylation patterns emerges, often resulting from changes in expression of histone modifiers, thus regulating chromatin accessibility and transcriptional activity [110,111,112]. Changes in H4K20me3, H4K16ac, H3K4me2/3, H3K9me2/3, H3K27me3, H3K9ac, and H3K18ac are the most frequently observed histone marks in BC [80,113,114,115,116,117,118,119]. Dysregulation of this intricate balance underscores the pivotal role of histone modifications in BC plasticity and aggressiveness.

### 4.1. Diagnostic Potential of Histone Modifications in BC Subtyping

Several studies underlined the potential of histone modification profiling for BC risk and subtype classification. For example, a positive relationship between H4K20me3 and status of hormone receptors (ER and PR), as well as a negative correlation between this histone mark and invasiveness has been reported by Yokoyama and colleagues [120]. More recently, low levels of H4K20me3 were found to be positively correlated with H4K16ac levels, and the combination of these two histone modifications (rather than the single alteration) is strongly associated with unfavorable prognosis in BC patients, highlighting the interplay between the two modifications and their potential as prognostic factors [121]. H3K9me3 levels are also related to ER status since they are particularly low in ER^+^ tumors compared to ER^−^ subtypes [80]; moreover, in ER^+^ BC patients, higher levels are associated with a more favorable prognosis [116].

A genome-wide histone modification study has revealed distinct binding patterns of H3K27me3 and H3K4me3 between human mammary epithelial cells and three breast cancer cell lines representing the luminal, HER2-enriched, and basal subtypes; while H3K27me3 and H3K4me3 genomic distribution is generally similar among subtypes, each cancer subtype exhibited thousands of unique, locus-specific binding events for the single modification [122]. Although highly variable, global H3K27me3 marks are reduced in BC, with the lowest levels usually observed in aggressive subtypes [117,118,119] and linked to chemoresistance [123]. At the molecular level, significantly lower H3K27me3 has been found in the promoters of *EZH2*, *P300*, and *SRC3* in HER2^+^ and basal-like BCs [119], thus suggesting that altered epigenetic regulation of these key epigenetic regulators may contribute to the aggressive behavior of these tumors. H3K4me2 and H3K4me3 levels also exhibit significant variability across BC subtypes. High H3K4me2 levels are indeed associated with better prognosis in BC patients and positively correlate with ER status [114]. On the other hand, high H3K4me3 levels are significantly associated with poor prognosis, regardless of ER status [114,115]. Furthermore, high H3K4me3 levels may contribute to receptor transcription, since H3K4me3 enrichment on the HER2 promoter in HER2-enriched BCs enhances promoter occupancy of the transcription factor AP-2, thus upregulating HER2 expression independently of gene amplification [124]. This evidence, coupled with the observation that H3K4me3 is regulated by the PI3K/AKT pathway [125], underscores the intricate interplay between signaling pathways and epigenetic mechanisms in BC development.

Wide H3K4me3 also positively correlates with H3K9ac, associated with HER2 status and poor prognosis in Ki67^high^ BC tissue samples [126]. H3K9ac is usually associated with active chromatin because of its ability to prevent H3K9 methylation. Moderate to low H3K9ac levels were observed in carcinomas of poorer prognostic subtypes [113]. Altered levels have been proven to be associated with aberrant gene regulation observed in TNBC (1016 modified gene promoters) and HER2^+^ (479 modified gene promoters) tumors [127,128].

### 4.2. Prognostic Implications of Histone Modifications

A hallmark alteration frequently observed in BC is the reduction and even the complete absence of H4K16ac [113,121,129], a flag typically associated with active gene transcription and maintenance of chromatin structure [130,131]. Loss of H4K16ac early occurs in BC, likely impairing DNA repair by preventing recruitment to DNA double strand breaks of the DNA damage response factor 53BP1 [113,132,133]. H4K16ac loss also leads to epigenetic silencing of the pro-apoptotic gene *TMS1*, further illustrating how disruption in histone acetylation may contribute to BC tumorigenesis [134].

Another example is represented by H3K9me2 and H3K9me3, whose levels are frequently lowered in BC; reduction in H3K9 methylation also appears to be progressive during cellular transformation. The biological relevance of this decrease is underlined by the observation that lessened H3K9me2 levels specifically activate *MYC* and *PAX3*, critical oncogenes in the early steps of tumorigenesis, as well as *bcl-2* and *pS2*, key cell survival genes [135,136,137]. Recent studies have also shown significant reduction in H3K9me3 levels at the promoters of immune checkpoint genes (*PD-1*, *CTLA-4*, *TIM-3*, and *LAG-3*) [80], suggesting a potential role for epigenetic regulation in immune evasion of BC cells.

Different alterations can coexist on the same histone residue, exerting synergistic or antagonistic effects on tumor cell behavior, thus contributing to BC plasticity. A representative example is the acetylation and trimethylation of H3K4. In vitro studies have revealed a global increase in both acetylation and methylation of H3K4, linked to active transcription and implicated in the regulation of genes involved in tumor progression [114,138]. Specifically, H3K4ac is associated with early cancer progression, while H3K4me3 correlates more with late-stage metastatic phenotypes.

Finally, dysregulation of H3K18ac, the marker of active transcription, may regulate gene expression programs driving oncogenic transformation, including genes promoting entry into the cell cycle, and inhibiting cell differentiation and the antiviral response. Accordingly, low H3K18ac levels in BC patients are associated with high tumor grade; conversely, high levels are positively correlated with hormone receptor, E-cadherin, and BRCA1 expression, negatively correlated with p53 and HER2 expression, and associated with a more favorable prognosis [113]. Such findings highlight the importance of undertaking genome-wide studies to explore the epigenetic reprogramming and the potential prognostic value of H3K18 hypoacetylation in BC.

All histone alterations in BC are largely driven by dysregulation of expression and/or activity of enzymes responsible for their establishment and removal (Table 2), thus contributing to dysregulated chromatin environment in cancer cells, evasion of apoptosis, and metastasis [101]. This is the case, for example, of EZH2 (responsible for H3K27 trimethylation), frequently overexpressed in aggressive BC subtypes and correlated with tumor proliferation, EMT, metastatic potential and poor prognosis [139,140], and of SUV4-20H2 (responsible for H4K20 methylation), whose expression is conversely reduced in BC and associated with invasive activity [120]. Several histone demethylases are dysregulated in BC as well. For instance, ER^+^ BCs exhibit overexpression of KDM4A and KDM4B; the two enzymes promote cancer growth and metastasis by targeting either the Notch1-NICD-dependent signaling (for KDM4A) or the estrogen signaling (for KDM4B) pathways [141,142].

Among HATs, p300 and KAT7 are both overexpressed in BC; the transcriptional coactivator p300 has been shown to be highly correlated with p53 and HIF-1a levels in invasive BCs [143], while KAT7 triggers cancer radioresistance by targeting the PI3K/AKT pathway [144]. Finally, HDAC isoenzymes 1, 2, and 3 are differentially expressed in BC: HDAC1 is highly expressed in hormone receptor positive BCs, correlates with better overall survival, and promotes proliferation of BC cells via activation of Snail/IL-8 signaling [145,146]; HDAC2 is highly expressed in hormone receptor negative BCs and correlates with tumor grade, lymph node status, and poor prognosis, as well as with several DNA-damage response genes [147]; HDAC3 also correlates with negative hormone receptor status and modulates apoptosis, cell cycle, metastasis, and angiogenesis [148].

**Table 2:** Dysregulation of histone modifier enzymes in BC.

	Enzyme	Substrate	Alteration	Association with Clinical Parameters	Refs
HMTs	EZH2	H3K27me1/2/3	↑ in invasive carcinoma and metastasis	-	[138,139]
	DOT1L	H3K79me1/me2/me3	↑ in ER^−^ BC	Poor survival and aggressiveness	[149,150]
	MLL2 (KMT2B)	H3K4me2/me3	↓ in BC	none	[151]
	MLL3 (KMT2C)	H3K4me1/2	↓ in hormone negative BC	-	[152]
	SUV4-20H2 (KMT5C)	H4K20me3	↓ in BC	-	[120]
	SETD1A	H3K4me	↑ in BC	-	[153,154]
	NSD3	H3K36me2/me3	↑ in BC	Worse overall and disease-free survival	[155]
	G9a (EHMT2)	H3K9me1/me2	↑ in BC	Poor outcome	[156]
HDMTs	KDM1A	H3K4me1/2 H3K9me1/2	↑ with DCIS and IDC advancement ↑ in basal-like BC	- Poor outcome	[157,158]
	KDM2B	H3K4me3 H3K36me2/3	↑ in TNBC	Poor prognosis/early relapse	[159,160]
	KDM3A	H3K9me1/2	↑ in BC	-	[135]
	KDM4A/B/C	H3K9me3, H3K36me2/3	↑ in ER^+^ BC	-	[141,142]
	KDM5A	H3K4me2/3	↑ in BC	Therapy resistance	[161]
	KDM5B	H3K4me1/2/3	↑ in HER2^+^ BC ↓ in basal-like BC	Poor outcome in ER^+^ BC	[162]
	KDM6B	H3K27me2/3	↓ in BC	Poor prognosis	[163]
HATs	p300	H3 (K14, K18, K23) H4 (K5, K8, K12)	↑ in BC	Grade, clinical stage, and tumor size, and recurrence	[143,164,165]
	CBP	H3 (K14, K18, K23) H4 (K5, K8, K12)	↑ in luminal A and B BC	ER and PR expression	[166]
	KAT2A	H3 (K9, K14, K18, K23)	↑ in BC	Tamoxifen-resistance	[166,167]
	KAT5	H2A, H3, H4	↓ in BC	-	[168]
	KAT6A	H3 (K9, K14) H4 (K5, K8, K12)	↑ in BC	ERα expression Worse clinical outcome	[169]
	KAT7	H4 (K5, K8, K12, K16)	↑ in BC	Worse clinical outcome	[144]
HDACs	HDAC1	H3, H4	↑ in BC	ER/HER2 expression	[146,170]
	HDAC2	H3K56, H4K16	↑ in poorly differentiated BC	HER2 status	[147]
	HDAC3	H3K9ac	↑ in poorly differentiated BC	ER/HER2 expression	[148]
	HDAC5	H3 (K9, K14) H4 (K5, K8, K12)	↑ in BC	-	[171]
	HDAC8	H3, H4	↑ in BC	-	[172,173]
	HDAC9	H3, H4	↑ in aggressive and tamoxifen-resistant BC	-	[174]
	HDAC11	H2A, H2B, H3, H4	↓ in basal-like BC	Unfavorable prognosis	[175]
	SIRT1	H3 (K9, K56) H4K16	↑ in BC	Tumor size and grade, lymph node, metastasis	[176]

BC: breast cancer; CBP: CREB-binding protein; DCIS: ductal carcinoma in situ; DOT1L: Disruptor of telomeric silencing 1-like; EHMT2: Euchromatic histone lysine methyltransferase 2; ER: estrogen receptor; EZH2: Enhancer of zeste homolog 2; HAT: Histone acetyltransferase; HDAC: Histone deacetylase; HDMT: Histone demethylase; HER2: epidermal growth factor receptor 2; HMT: Histone methyltransferase; KAT: Lysine acetyltransferase; IDC: invasive ductal carcinoma; KDM: Lysine demethylase; KMT: Lysine methyltransferase; MLL: Mixed lineage leukemia protein; NSD3: Nuclear receptor binding SET domain protein 3; p300: EP300 (E1A binding protein p300); PR: progesterone receptor; SETD1A: SET domain containing 1A, histone lysine methyltransferase; SIRT1: Sirtuin 1 (NAD-dependent deacetylase); SUV4-20H2: Suppressor of variegation 4-20 homolog 2; TNBC: triple negative breast cancer. ↑ denotes upregulated; ↓ denotes downregulated; - not indicated.

### 4.3. Therapeutic Implications of Histone Modifications in BC

Because epigenetic changes are reversible and histone modifiers are druggable, targeting them will provide unique opportunity for pharmacological intervention through inhibitors that represent a novel class of anti-cancer drugs [177]. Clinical success of epi-drugs in solid tumors (including BC) has, however, so far been limited, given the inherent cell specificity of epigenetic modifications [178].

Among the inhibitors developed, HDAC inhibitors stand out as some of the most effective agents with significant therapeutic potential, and some are currently undergoing clinical validation [179,180]. They target HDACs to promote hyperacetylation, thereby activating pathways involved in cell cycle arrest, apoptosis, and reversal of EMT. One of the most extensively studied pan-HDAC inhibitor is Vorinostat (suberoylanilide hydroxamic acid, SAHA), which received Food and Drug Administration (FDA) approval in 2006 for treatment of cutaneous T-cell lymphoma [181,182]. Studies in preclinical models have proven its ability to induce cell cycle arrest and apoptosis [183,184]. For instance, SAHA increased acetylation on H3 and H4 histones, dose-dependently inhibited proliferation, and induced autophagic cell death in tamoxifen-resistant MCF-7 cells; moreover, SAHA treatment was able to reduce tumor growth and weight in mice bearing tamoxifen-resistant MCF-7 xenografts [184]. At clinical level, SAHA monotherapy demonstrates tolerable toxicity, but its therapeutic response remains limited, indicating that combination therapy with other agents may offer more substantial benefits; combined therapy with tamoxifen has shown, for example, promising anti-tumor effects in ER^+^ BC patients with advanced disease [185].

Panobinostat (LBH589), another pan-HDAC inhibitor, also possesses potent inhibitory activity against hematological malignancies and treatment-resistant solid tumors. Several investigations have revealed that Panobinostat effectively impacts TNBC cells and reduces tumorigenesis in vivo by upregulating anti-proliferative genes, tumor suppressors, and epithelial markers [186,187]. It also significantly reduces invasive capacity of TNBC cells, suggesting its potential application in aggressive BCs less responsive to hormonal therapies [188], although a phase II clinical trial (NCT00777049) was unable to determine the efficacy of this drug, maybe because of small sample size.

Preclinical data led to multicenter, randomized, double-blind, placebo-controlled phase III trials, but unsuccessfully [189]. For example, the ENCORE301 phase II study reported increased progression-free and overall survival in postmenopausal women with advanced HR^+^ and HER2^−^ BC, not responsive to endocrine therapy, and treated with entinostat (HDAC inhibitor) plus exemestane (aromatase inhibitor) [190]; however, the E2112 phase III study did not confirm these results since no improvement in survival was documented [191]. Conversely, the Chinese ACE phase III study reported a significant increase in progression-free survival after treatment with tucidinostat plus exemestane [192]. The apparent discrepancy could be explained by considering that (i) the study is restricted to a single racial patient cohort, which may limit the generalizability of the results; (ii) quality of life data is absent; and (iii) the observed efficacy may be biased due to the lower prevalence of prior endocrine therapy in the cohort compared to other HDAC inhibitor trials. Because of these limitations, no HDAC inhibitor has been approved for BC treatment up to now, but further preclinical and clinical investigation of HDAC inhibition, especially in combination therapies, is warranted.

Another class of epi-drugs gaining attention in BC research involves inhibitors directly or indirectly targeting EZH2. DZNep (3-deazaneplanocin A hydrochloride) is a *S*-adenosylhomocysteine hydrolase (SAAH) inhibitor depleting cellular levels of PRC2 components (EZH2, SUZ12, and EED), suppressing histone methylation, and inducing selective apoptosis in BC cell lines and primary mammary tumors [193,194,195]. Notably, DZNep has been shown to overcome tamoxifen-resistance in BC cells by inhibiting another methyltransferase (NSD2) rather than EZH2 itself [196].

A direct and selective drug is tazemetostat (EPZ-6438), the first EZH2 inhibitor to receive FDA approval for treatment of adults and adolescents aged ≥ 16 years with locally advanced or metastatic epithelioid sarcoma [197]; it is a competitive inhibitor showing promising results in phase I and II clinical trials for certain cancer types [198,199]. Currently ongoing clinical studies for BC are not available, although some promising preclinical studies have emerged, particularly in the context of TNBC: EPZ-6438 treatment significantly reduced H3K27me3 levels, increased ERα expression in TNBC cell lines, and promoted inhibition of cell growth and sensitivity to tamoxifen in murine models [200].

In conclusion, epi-drugs hold promise for BC treatment, but their clinical application is challenging. While monotherapy often yields limited efficacy and significant side effects, combination therapies show potential for improved outcomes by synergistically targeting multiple cancer pathways [201]. Further research is essential for optimizing the use of epi-drugs in specific BC subtypes.

## 5. Non-Coding RNAs in BC

The epigenetic landscape is also intricately regulated by non-coding RNAs, also referred to as the “dark matter” of the genome [202], among which long non-coding RNAs (lncRNAs) and microRNAs (miRNAs) have emerged as key players in regulating chromatin structure, gene expression, and cellular phenotype [203]. LncRNAs are non-coding transcripts (>200 nucleotides) regulating gene expression at transcriptional or post-transcriptional level [204]. Their function as chromatin regulators lies on the ability to interact with and recruit chromatin-modifying enzymes to specific chromatin loci; for instance, KCNQ1OT1 binds to PRC2 and EZH2, leading to H3K27 trimethylation [205]. The Hox transcript antisense intergenic RNA (HOTAIR) is a master lncRNA regulator as it scaffolds repressive histone modifying enzymes; its 5′ domain binds to and modulates PRC2/EZH2 activity (involved in H3K27me3 formation), while the 3′ domain recruits a repressive complex constituted by the lysine demethylase LSD1 and the deacetylases CoREST/REST (involved in H3K4 demethylation) [206]. miRNAs are short (~22 nucleotides) RNAs that post-transcriptionally downregulate gene expression by binding to the 3′ untranslated region (3′UTR) of target mRNAs, thus inhibiting translation or inducing direct degradation [207]. These non-coding RNAs can inhibit gene expression also by interacting with other regions, such as the 5′UTR, the coding sequence, and even gene promoters [208]. A large body of literature data highlighted the interplay between miRNAs and epigenetic enzyme modifiers with far-reaching implications for human health and disease; indeed, the delicate equilibrium of methylation and acetylation reactions is susceptible to fine-tuned regulation by miRNAs. For example, DNA methylation is controlled by several miRNAs, including miR-29b, miR-126, and miR-377, which directly target different members of the DNMT and TET families [209,210]; similarly, miR-34a and miR-148a, respectively, control HDAC1 and HDAC5 expression, thus regulating H3K acetylation [211,212]. Additionally, miRNAs modulate both histone methylation and demethylation: for example, miR-941 [213] and miR-137 [214] directly target several KDM enzymes, while miR-126, miR-124, let-7, and miR-combo (a combination of miR-1, miR-133a, miR-208a, and miR-499) repress EZH2 expression [215,216,217]. Finally, miRNAs have also been reported to impact epigenetic landscape during embryonic development by regulating the ATP-dependent chromatin remodelers (ACRs) complexes; for instance, miR-302 is highly expressed in human embryonic stem cells, where it promotes self-renewal and pluripotency by directly downregulating the expression of BAF53a and BAF170 proteins that are part of the SWI/SNF (switching defective/sucrose nonfermenting) complex, a common ACR acting as master regulators of nucleosome positioning and occupancy in chromatin [218,219].

In the intricate landscape of epigenetic regulation, ncRNAs introduce an additional layer of gene expression control as they are not only subjected to epigenetic regulation themselves but also actively influence the epigenetic machinery. These ncRNAs are capable of recruiting and interacting with histone modifiers, thereby modulating chromatin structure and transcriptional activity; furthermore, they also regulate DNMT activity, contributing to establishment and maintenance of DNA methylation patterns. Through these interactions, the ncRNAs orchestrate epigenetic changes that directly and indirectly impact the expression of both coding and non-coding transcripts [220].

### 5.1. Diagnostic and Prognostic Potential of ncRNAs

Among lncRNAs, HOTAIR is the most extensively studied in cancer biology; it exerts oncogenic activity by exerting repressive effects on chromatin, thus silencing genes involved in cell differentiation and maintaining stem cells characteristics [221,222]. For instance, by recruiting PRC2 to the IkBα promoter, HOTAIR suppresses its expression and activates NF-κB signaling, thereby facilitating the maintenance of BC stem cell self-renewal [223]. Aberrant HOTAIR overexpression has consistently been found in primary BC tumors, particularly those with high metastatic potential and poor clinical outcomes [224]. Mechanistically, HOTAIR overexpression drives tumor progression by recruiting PCR2 and inducing H3K27me3 deposition on the promoters of suppressor genes; therefore, HOTAIR expression positively correlates with BC EMT transition, invasiveness and metastatic potential [225].

Unlike HOTAIR, the antidifferentiation noncoding RNA (ANCR) acts as a tumor suppressor. ANCR inhibits BC cell migration and invasion acting as a crucial negative regulator of EMT, mainly through affecting EZH2 stability. This lncRNA, indeed, interacts with EZH2 facilitating CDK1 binding with this methyltransferase, thus promoting its degradation. The expression of ANCR in BC has been found lower in ER^−^ subtypes compared with ER^+^ ones and much lower in HER2^−^ BC with respect to HER2^+^ subtypes. Therefore, ANCR may represent a potential biomarker for highly aggressive and malignant BCs [226].

The regulator of reprogramming (ROR) lncRNA is frequently altered in solid tumors (such as pancreatic, hepatocellular, endometrial, nasopharyngeal, and breast cancers) [227]. Its tumor-promoting effect is closely linked to regulation of multiple signaling pathways, although its main function appears to correlate with maintenance of stem cell pluripotency. By recruiting MLL1 (lysine methyltransferase 2A, KMT2A), this lncRNA stimulates H3K4 methylation and upregulation of TIMP3 (inhibitor of metalloproteinases 3), with subsequent induction of migration and inhibition of apoptotic death, thus contributing to BC invasiveness [228].

Distinct miRNA profiles have been associated with different BC subtypes, although they profoundly vary within subtypes due to BC heterogeneity. miRNAs can act either as oncogenes or tumor suppressors, thus exerting specific functions able to affect proliferation, apoptosis, invasion, metastasis, and tumor stemness. Several oncomiRs have been identified so far, including miR-10 [229], mir-17-5p [230], miR-183 [231], miR-210 [232], and miR-221/222 [233]; they directly affect specific pathways, such as the induction of apoptosis by miR-17-5p (via inhibition of STAT3 and upregulation of p53) [234], or can influence the tumor microenvironment [235]. Likewise, tumor suppressor miRNAs, including miR-26b [236], miR-124-3p [237], miR-126 [238,239], miR-203 [240], and miR-205 [241], mainly act by silencing oncogenes that promote breast tumorigenesis. Furthermore, the effects of certain miRNAs appear to be context-specific; this is the case, for example, of miR-125b that inhibits cell growth in luminal and HER2-enriched BCs, while stimulating the growth of TNBC cells [242,243].

Beyond the few examples discussed, the literature extensively covers the diverse functions of miRNAs as epigenetic regulators in BC, as detailed in several reviews [244,245]. Crucially, miRNAs also directly modulate epigenetic modifiers [246,247], introducing a further regulatory layer to the complex network of epigenetic gene expression control, which is summarized in Table 3.

The miRNA-epigenetic feedback loops may also have dual functions depending on the context and molecular target. A notable example is represented by miR-22, which can play opposite roles in different scenarios; it suppresses tumorigenesis and favorably contributes to radiotherapy sensitivity by modulating SIRT1 [255], but, conversely, it promotes EMT and tumor stemness by indirectly acting on TET family enzymes [261].

Moreover, miRNA/target gene reciprocal regulations are established. For example, miR-200b, belonging to the miR-200 family (whose members are downregulated in BC) [262], is repressed in TNBC as a consequence of promoter hypermethylation triggered by DNMT3A, which is itself a direct target of miR-200b [263]. Another example is represented by the DNMT1/miR-148a and miR-152 circuit [273]: DNMT1 catalyzes promoter hypermethylation and silencing of the two miRNAs in both BC cells and tissues, thus upregulating IGF-IR and IRS1 (which are also targeted by miR-148a and miR-152) and promoting tumor growth and angiogenesis [272].

The scenario is further complicated by the evidence that miRNAs can belong to specific “feed forward loops”, a gene network where one or more transcription factors regulate the expression of a specific miRNA, and both independently regulate a set of target genes [282]. Recently, we have proposed the existence of a feed forward loop, whose dysregulation contributes to BC progression and acquisition of stemness properties. Specifically, downregulation of transcription factors and subsequent decrease in miR-126 levels leads to increased expression of HOTAIR, EZH2, and DNMT1, thus impairing the epigenetic control of genes involved in differentiation, development, and tumor suppression [239].

In conclusion, ncRNAs represent key elements in regulation of cancer biology, not only for their role as regulators of gene expression but also as mediators of epigenetic changes. Their capacity to modulate complex mechanisms, such as DNA methylation, histone modifications, and mRNA stability, makes them crucial in cancer pathogenesis and may provide innovative opportunities for developing personalized treatments in oncology.

### 5.2. Therapeutic Potential of ncRNAs

HOTAIR overexpression has been implicated in radiotherapy resistance, as proven by in vitro and in vivo studies [283]; specifically, it recruits EZH2 to the *MYC* promoter, thus regulating genes involved in DNA repair and survival pathways [283]. Collectively, these findings emphasize the multifaceted role of HOTAIR in epigenetic regulation, tumor metastasis, radioresistance, and stem cell maintenance in BC, rendering it a potential prognostic factor and a suitable therapeutic target. Targeting approaches, using HOTAIR mutants lacking the EZH2-interacting domain, have indeed been found to inhibit EMT, thus allowing for the rescue of a more differentiated phenotype in hepatocarcinoma cells [221]. Also, pharmacological approaches have been proven to be useful; for example, treatment of BC cells with metformin (a hypoglycemic drug with anticancer properties) promotes methylation and downregulation of HOTAIR expression, reverting the EMT properties of tumor cells [221]

H19 is an imprinting lncRNA exclusively transcribed from the maternally inherited allele [284]. It is overexpressed in either DCIS or invasive BC, with respect to normal breast tissues, and plays crucial roles in proliferation, metastasis, chemoresistance, and stem cell properties [285,286]. Recent studies have underscored the role of H19 also in BC resistance to radiotherapy and endocrine therapy [287,288]. Just an example, H19 expression is considerably elevated in both tamoxifen-resistant cell lines and tumor tissues and its silencing can inhibit autophagy and restore sensitivity to tamoxifen [286]. At molecular level, H19 has been shown to inhibit SAHH, with subsequent accumulation of *S*-adenosylhomocysteine and inhibition of SAM-dependent methyltransferases [289]; therefore, the presence of H19 reduces the DNMT3B-mediated methylation of *Beclin1* promoter, whose activation leads to induction of autophagy and acquisition of tamoxifen-resistance [287].

Because of their regulatory potential, which directly and indirectly (by modulating epigenetic enzymes) impacts gene expression, miRNAs represent a promising field for developing innovative therapeutic strategies. Indeed, great efforts are being focused on finding new therapeutic strategies, which directly target miRNAs (miRNA-based therapies with specific oligonucleotides) or restore the expression of epigenetically silenced miRNAs, for improving cancer treatment [290,291]. For instance, reintroduction of miR-34a into BC cells has been shown to suppress growth, migration, and invasion by negatively regulating *E2F3*, *CD44*, and *SIRT1* target genes; in mouse models, liposomal nanoparticles containing miR-34a efficaciously inhibit tumor growth and increases survival, with no evidence of systemic toxicity [292]. Reintroduction of miR-34a is also able to suppress the aggressive phenotype of TNBC cells and enhance the sensitivity to dasatinib-based chemotherapy [293]. In preclinical studies, inhibition of miR-10b expression, a prometastatic microRNA, by a specific antagomiR, leads to significant reduction of lung metastasis formation [294]. In this context, several epi-drugs can modulate miRNA expression. Treatment of BC cells with SAHA impairs proliferation, invasion, and migration, a phenomenon attributed to the induction of miR-200c expression [295]. Entinostat has demonstrated significant potential in treatment of BC, particularly against ER^+^ and HER2-enriched subtypes [296]; the drug upregulates miR-125a, miR-125b, and miR-205, all of which are involved in apoptosis induction of BC cells and able to downregulate HER2 and HER3 expression [297].

While miRNA-based therapeutics show promise, they remain largely confined to in vitro and preliminary in vivo models. Several hurdles, including oligonucleotide stability, targeted delivery to tumor tissues, and potential off-target effects, necessitate rigorous investigation. Bridging the gap between observed preclinical efficacy and clinically translatable therapies hinges on addressing these critical challenges.

## 6. Conclusions

The epigenetic landscape of BC is a complex interplay of genetic, environmental, and molecular factors that contribute to the disease’s remarkable heterogeneity, progression, and treatment response.

**Figure 3:**
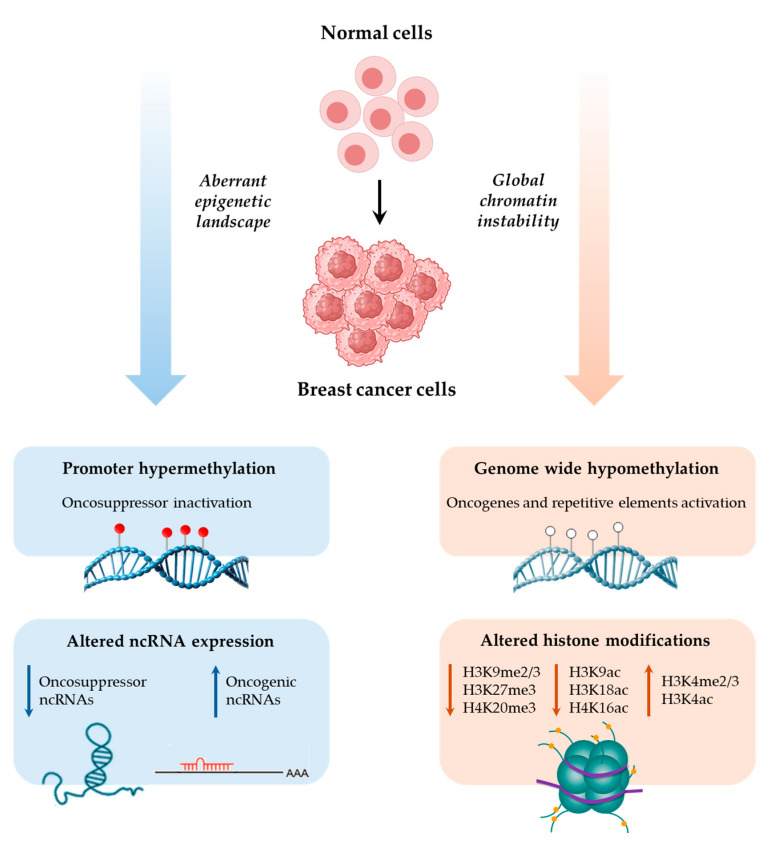
Schematic representation of epigenetic changes observed in the transition from normal to BC cells. Left part depicts processes leading to the aberrant epigenetic landscape of cancer cells; right part depicts processes leading to genomic instability. ↑ and ↓ in each box denote increase or decrease, respectively.

While not comprehensive, this review underscores the critical contribution of epigenetics—including DNA methylation, histone modifications, and ncRNA regulation—to BC development and progression (Figure 3). Further investigation into the interactions of these epigenetic players will deepen our understanding of BC biology and reveal potential therapeutic targets for patient stratification and monitoring.

Although significant advancements have been made in developing epigenetic therapies, their clinical application remains limited, and to date, no epi-drugs have been approved for BC management. Failure in clinical settings lies in several limitations of epigenetic therapy. First, epi-drugs target cancer cells to “reprogram” their biology or re-sensitize them to conventional therapies for improving responses; this global effect necessarily requires the development of novel endpoints and read outs for efficacy evaluation. Second, epi-drugs have widespread effects, necessitating a comprehensive understanding of their action on healthy cells, tumor microenvironment, and immune system to minimize “off-target” consequences. Finally, intra-tumoral heterogeneity and cancer cell plasticity must be considered as this allows cancer cells to rapidly adapt through genetic and epigenetic changes. In conclusion, future research is required for harnessing the power of epigenetics, enabling precision medicine approaches that could significantly ameliorate prognosis, treatment, and outcomes of BC patients.

## Figures and Tables

**Table 1:** Dysregulation of DNA methylation in some genes involved BC onset and progression.

Gene	Main Findings	Clinical Relevance	Ref.
**Hypermethylated genes**			
*BRCA1*	Positively related to tumor grade, ER and PR status	Predictive	[45]
	No correlation with clinicopathological features	-	[46]
	Associated with better prognosis and chemotherapy efficacy	Prognostic	[47]
	Related to grade, Ki67, and HER2 levels	Diagnostic/prognostic	[48]
*MGMT*	Related to lymph node involvement, grade and stage, and ER and PR loss	Therapy response	[49]
	Associated with TNBC	Therapy response	[50]
*E-cadherin*	Positively correlated with ER expression, lymph node metastasis, and poor overall and disease-free survival	Prognostic	[51,52]
*RARβ2*	Hypermethylation marks the transition from proliferative epithelial hyperplasia towards atypia, occurring especially during transition from DH to ADH	Risk biomarker	[53,54,55]
*Cyclin D2*	Related to poor prognosis	Diagnostic/prognostic	[56]
*RASSF1A*	Related to tumor size, ER and PR status, immunohistochemical subtype	Prognostic Therapy response	[57]
	Hypermethylation marks the transition from normal epithelia towards DH	Risk biomarker	[54]
*CDKN2A* *(p14ARF/p16INK4a)*	*p14ARF* methylation related to peritumoral vessel involvement, p53 mutations and PR^−^	Prognostic	[58]
	*p16INK4a* methylation negatively associated with ER, PR, and HER2 expression	Diagnostic	[59]
*ERα*	Related to resistance to endocrine therapy	Therapy response	[60,61]
*PTEN*	Related to tumor size, histologic grade, ER and HER2 status, and disease-free and overall survival	Prognostic	[62,63,64]
*APC*	Hypermethylation marks the transition from healthy tissue to benign lesions to BC; no correlation with clinicopathological features	Risk biomarker	[55]
	Related to chemotherapy, distant metastasis, and overall survival	Prognostic	[65]
**Hypomethylated genes**			
*LINE-1*	Associated with tumor stage, lymph node metastasis, older age, distant recurrence, and disease-free and overall survival	Prognostic	[66]
*CXCR4*	Correlated with tumor stage and size, histological grade, lymph node status, metastasis, and death	Prognostic	[67]

ADH: atypical ductal hyperplasia; APC: Adenomatous Polyposis Coli; BC: breast cancer; BRCA1: Breast Cancer Type 1 Susceptibility Protein; CDKN2A (p14ARF/p16INK4a): Cyclin-Dependent Kinase Inhibitor 2A; CXCR4: C-X-C Motif Chemokine Receptor 4; Cyclin D2 (CCND2): G1/S-Specific Cyclin-D2; DH: ductal hyperplasia; E-cadherin (CDH1): Epithelial Cadherin; ERα: Estrogen Receptor Alpha; HER2: epidermal growth factor receptor 2; LINE-1: Long Interspersed Nuclear Element-1; MGMT: O-6-Methylguanine-DNA Methyltransferase; PTEN: Phosphatase and Tensin Homolog; PR: progesterone receptor; RARβ2: Retinoic Acid Receptor Beta 2; RASSF1A: Ras Association Domain Family Member 1; TNBC: triple negative breast cancer.

**Table 3:** Dysregulation of some miRNAs involved in epigenetic regulation in BC.

	miRNAs (*target*)	Biological Role	Dysregulation	Refs
Proliferation	miR-17-5p (*KAT13B*)	Inhibition of proliferation	↓ in BC	[230]
	miR-137 (*KDM5B*)	Inhibition of proliferation and migration		[248]
	miR-138 (*KDM5C*)			
	miR-142-5p (*DNMT1*)			[249,250]
	miR-143 (*DNMT3A*)			[251]
	miR-381-3p (*SETDB1*)			[252]
	miR-185 (*DNMT1*)	Inhibition of proliferation by indirectly up-regulating BRCA1 expression	↓ in TNBC	[253]
	miR-29a (*TET1*)	Increase in proliferation, migration, and EMT; negatively correlated with poor survival	↑ in ER^−^ BC	[254]
EMT, invasion, migration, metastasis	miR-22 (*SIRT1*)	Inhibition of tumorigenesis and improvement of radiosensitivity	↓ in BC	[255]
	miR-126 (*EZH2*, *DNMT1*)	Inhibition of proliferation, EMT, invasion, and metastatic potential		[239,256,257]
	miR-138 (*KDM6B*)	Inhibition of EMT and invasion; associated with lymph node metastasis, TNM stage, and poor prognosis		[258]
	miR-502 (*KMT5A*)	Inhibition of proliferation, invasion, migration, and EMT		[259]
	miR-765 (*EZH1*)	Inhibition of proliferative, migratory and invasive abilities; related to tumor stage, metastasis, metastasis, and poor prognosis		[260]
	miR-22 (*TET1/2/3*)	It indirectly targets TET family members by antagonizing miR-200b; related to stemness, EMT, invasion, metastasis, and poor clinical outcomes		[261]
	miR-200b (*DNMT3A*)	Inhibition of EMT	↓ in TNBC	[262,263]
	miR-340 (*EZH2*)	Inhibition of cell growth, invasion and migration, and induction of apoptosis		[264]
	miR-770-5p (*DNMT3A*)	Inhibition of EMT and invasion		[265]
	miR-29 (*SUV420H2*)	Stimulation of EMT, migration, and invasion	↑ in BC stem cells	[266]
	miR-29a (*TET1*)	Stimulation of EMT, proliferation, and migration	↑ in ER^−^ BC	[254]
	miR-25, miR-93, miR-106b (*P300*)	Stimulation of invasion, migration, and EMT	↑ in BC	[267]
Therapy resistance	miR-17, miR-20b (*KAT13B*)	Stimulation of chemosensitivity	↓ in taxol-resistant cells	[268]
	miR-22 (*SIRT1*)	Inhibition of tumorigenesis and stimulation of radiosensitivity	↓ in BC	[254]
	miR-34a (*HDAC1*, *HDAC7*)	Stimulation of therapy sensitivity; negatively correlated with tumor grade and stage		[269]
	miR-486 (*KDM5B*)	Involved in DNA damage repair and radiosensitivity		[270]
	miR-10b (*HDAC4*)	Associated with invasiveness, migration, and tamoxifen-resistance	↑ in tamoxifen-resistant cells	[271]
Angiogenesis	miR-148a, miR-152 (*DNMT1*)	Inhibition of tumor growth and angiogenesis by down-regulating IGF-IR and IRS1	↓ in BC	[272,273]
Apoptosis	miR-26a (*EZH2*)	Inhibition of proliferation and induction of apoptosis		[274]
	miR-125a-5p (*HDAC5*)	Stimulation of apoptosis in BC stem cells by targeting apoptosis-related genes		[242,275]
	miR-590-3p (*SIRT1*)	Stimulation of apoptosis		[276]
Stemness	miR-7 (*SETDB1*)	Reverses the EMT of BC stem cells by downregulating the STAT3 pathway	↓ in BC stem cells	[277]
	miR-34a (*SIRT1*)	Inhibition of stemness markers		[278]
	miR-200b (*SUZ12*)	Regulation of E-cadherin expression and stemness		[279]
	miR-221, miR-222 (*DNMT3B*)	Stimulation of expression of pluripotency-associated genes (*Nanog*, *Oct 3/4*)	↑ in BC stem cells	[280]
Estrogen signaling	miR-491-5p (*KDM4B*)	Inhibition of estrogen signaling and estrogen-stimulated proliferation	↓ in ERα^+^ BC	[281]

BC: breast cancer; EMT: epithelial–mesenchymal transition; ER: estrogen receptor; TET: ten-eleven translocation; TNBC: triple negative breast cancer. ↑ denotes upregulated; ↓ denotes downregulated.

## Data Availability

Data sharing is not applicable to this article as no datasets were generated or analyzed during this current study.
